# Ubiquitylation of cyclin C by HACE1 regulates cisplatin‐associated sensitivity in gastric cancer

**DOI:** 10.1002/ctm2.770

**Published:** 2022-03-28

**Authors:** Hong‐yue Jiang, Ying‐ling Chen, Xing‐xing Xu, Chuan‐yin Li, Yun Chen, Dong‐ping Li, Xiao‐qing Zeng, Hong Gao

**Affiliations:** ^1^ Department of Gastroenterology and Hepatology Zhongshan Hospital Fudan University Shanghai China; ^2^ State Key Laboratory of Molecular Biology CAS Center for Excellence in Molecular Cell Science Innovation Center for Cell Signaling Network Shanghai Institute of Biochemistry and Cell Biology Chinese Academy of Sciences Shanghai China; ^3^ University of Chinese Academy of Sciences Beijing China; ^4^ Evidence‐based Medicine Center of Fudan University Shanghai China

**Keywords:** cisplatin, cyclin C, HACE1, mitochondrial stability, ubiquitylation

## Abstract

**Background:**

Cyclin C (*CCNC*) was reported to take part in regulating mitochondria‐derived oxidative stress under cisplatin stimulation. However, its effect in gastric cancer is unknown. This study aimed to investigate the role of cyclin C and its ubiquitylation in regulating cisplatin resistance in gastric cancer.

**Methods:**

The interaction between HECT domain and ankyrin repeat‐containing E3 ubiquitin‐protein ligase 1 (HACE1) and cyclin C was investigated by GST pull‐down assay, co‐immunoprecipitation and ubiquitylation assay. Mitochondria‐derived oxidative stress was studied by MitoSOX Red assay, seahorse assay and mitochondrial membrane potential measurement. Cyclin C‐associated cisplatin resistance was studied in vivo via xenograft.

**Results:**

HACE1 catalysed the ubiquitylation of cyclin C by adding Lys11‐linked ubiquitin chains when cyclin C translocates to cytoplasm induced by cisplatin treatment. The ubiquitin‐modified cyclin C then anchor at mitochondira, which induced mitochondrial fission and ROS synthesis. Depleting *CCNC* or mutation on the ubiquitylation sites decreased mitochondrial ROS production and reduced cell apoptosis under cisplatin treatment. Xenograft study showed that disrupting cyclin C ubiquitylation by HACE1 conferred impairing cell apoptosis response upon cisplatin administration.

**Conclusions:**

Cyclin C is a newly identified substrate of HACE1 E3 ligase. HACE1‐mediated ubiquitylation of cyclin C sheds light on a better understanding of cisplatin‐associated resistance in gastric cancer patients. Ubiquitylation of cyclin C by HACE1 regulates cisplatin‐associated sensitivity in gastric cancer. With cisplatin‐induced nuclear–mitochondrial translocation of cyclin C, its ubiquitylation by HACE1 increased mitochondrial fission and mitochondrial‐derived oxidative stress, leading to cell apoptosis.

## Graphical abstract

- Cyclin C is a new substrate of E3 ubiquitin ligase HACE1.
- The ubiquitylation modification of cyclin C is in the non‐proteolytic K11 ubiquitin linkage form and critical for the cisplatin‐induced nuclear–mitochondrial translocation of cyclin C.
- Ubiquitylation of cyclin C by HACE1 regulates gastric cancer cell cisplatin sensitivity via modulating mitochondrial stability.

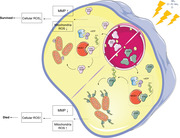

## 1 BACKGROUND

Gastric cancer is the second leading cause of cancer‐related death.^1^ Even though many achievements have been made in early diagnosis and chemotherapy for gastric cancer, the emerging resistance to therapeutic drugs, such as cisplatin, becomes a big challenge for gastric cancer treatment.^2^, ^3^ Therefore, understanding the mechanism of cisplatin resistance is urgently needed to find novel approaches to tackle chemotherapeutic resistance and improve patient prognosis.

Cyclin C (gene name: *CCNC*) belongs to the human cyclin family and is an important transcriptional factor.^4^ Cyclin C, together with cyclin‐dependent kinase 8 (CDK8), mediator complex subunit (Med) 12 and 13, reversibly forms a regulatory subcomplex called CDK8‐dependent kinase module (CKM),^5^ which inhibits protein transcription by phosphorylation of the C‐terminal domain of RNA polymerase II.^6^ Another study also reported that cyclin C plays a role in regulating mitochondrial dynamics.^7^ In response to cisplatin, cyclin C translocates to mitochondria and takes part in mitochondrial fission, featured by increased mitochondrial fragmentation, which leads to an increase in oxidative stress and cell apoptosis in both mammalian cells and yeast.^7^, ^8^, ^9^, ^10^ So far, little is known about the role of cyclin C in regulating mitochondrial dynamics in response to cisplatin stimulation in gastric cancer. And whether it affects cisplatin resistance in gastric cancer is poorly defined.

Protein ubiquitylation, one of posttranslational modification (PTM), has the potential to regulate the function of cyclin C. To initiate ubiquitylation modification, an E3 ligase is required to catalyse the addition of ubiquitin (Ub) to the substrates. HECT domain and ankyrin repeat‐containing E3 ubiquitin‐protein ligase 1 (HACE1) is one of the well‐recognised E3 ligases that acts as a tumour suppressor in different cancers, including gastric cancer.^11^, ^12^, ^13^, ^14^ Our previous study revealed that HACE1 was pravelently downregulated in gastric cancer, and its reduced protein level related to a poor prognosis, so we are interested to know whether HACE1 regualtes cisplatin resistance, thereby, affecting the prognosis.^15^

And we recently found that HACE1 and cyclin C could interact with each other in vivo and in vitro. So it was interesting to further investigate whether HACE1 regualtes cisplatin response. In this study, we identified that in gastric cancer, HACE1 catalyses the ubiquitylation modification of cyclin C when it translocates to cytoplasm under the stimulation by cisplatin treatment. The K11 ubiquitin chain‐modified cyclin C then induces the mitochondrial functional dysregulation, which leads to enhanced mitochondrial fission and reactive oxygen species (ROS) synthesis. Disruption of HACE1 and cyclin C interaction by *CCNC* deletion or site mutation mitigated the mitochondrial ROS production as well as the cisplatin‐induced apoptosis in gastric cancer cells. Thus, HACE1‐mediated ubiquitylation modification of cyclin C plays a role in regulating cell response to cisplatin treatment in gastric cancer.

## 2 METHODS AND MATERIALS

### 2.1 Immunoblotting

For the immunoblotting in the present study, the following antibodies were adopted: HA (H6908, Sigma‐Aldrich, 1:4 000), Flag (20543‐1‐AP, Proteintech, 1:1000), His (66005‐1‐lg, Proteintech, 1:2000), glutathione (GST; HRP‐66001, Proteintech, 1:5000), Myc (sc‐40, Santa Cruz, 1:1000), Lamin B1 (66095‐1‐lg, Proteintech, 1:5000), HACE1 (ab133637, Abcam, Cambridge, UK, 1:2000), cyclin C (A301‐989A, Bethyl, 1:2000), Ub (sc‐8017 HRP, Santa Cruz, 1:1000), tubulin (10094‐1‐AP, Proteintech, 1:1000), Hsp90b (7411, Cell Signaling Technology, Danvers, MA, USA, 1:1000), mitofusin‐1 (14739, CST, 1:1000), Drp1 (ab56788, Abcam, 1:1000), COX4 (11242‐1‐AP, Proteintech, 1:5000) and GAPDH (60004‐1‐AP, Proteintech, 1:4000).

### 2.2 Cell culture and transfection

The Stem Cell Bank in the Chinese Academy of Sciences kindly provided MKN28, MGC‐803 and HGC27 cell lines. HEK‐293FT cells were transfected with the indicated plasmids using polyethyleneimine (Sigma‐Aldrich), while the transfection of HGC27 cells was done with the Attractene Transfection Reagent (Qiagen) according to the manufacturer's instructions. HEK‐293FT and HGC27‐based *HACE1* or *CCNC* knockout cell lines were generated by the CRISPR/Cas9 system.^16^ Pairs of sgRNAs targeting *CCNC* and *HACE1* were designed, as reported previously^16^ and are listed below (target sequences are lowercase):

*HACE1*: (1) 5′‐CACCgcaactccacggtgcgcgcg‐3′, (2) 5′‐AAACcgcgcgcaccgtggagttgc‐3′;
*CCNC*: (1) 5′‐CACCGcttgaaatataccgtagcag‐3′, (2) 5′‐AAACctgctacggtatatttcaagC‐3′

### 2.3 Protein purification and GST pull‐down assay

The cDNA encoding HACE1 was cloned into pGEX4T‐1 to yield pGEX4T1‐HACE1. The cDNA of *CCNC* was cloned into pET22b (+) and pSJ8 vector (pET21a derivative, MBP‐His fusion vector) for expressing cyclin C in an *Escherichia coli* BL21 expression strain. GST, GST‐HACE1 and GST‐HACE1‐truncated proteins were purified as described previously.^11^ MBP‐His‐tagged cyclin C proteins were purified using Ni‐NTA agarose beads (Qiagen, Venlo, Netherlands) following the manufacturer's protocols. Purified cyclin C‐MBP‐His and GST‐HACE1 proteins were incubated with gentle end‐over‐end mixing for 6 h at 4°C in the pull‐down buffer (50 mM Tris‐Cl, 200 mM NaCl, 1 mM EDTA, 1% NP‐40, 1 mM DTT, 10 mM MgCl_2_, 10 μg/ml BSA, pH = 8.0). The complexes were washed with the pull‐down buffer and subjected to the immunoblotting assay.

### 2.4 Mitochondria isolation

According to the manufacturer's instructions, mitochondrion‐enriched subcellular fractions were prepared using the Mitochondria Isolation Kit for cultured cells (#89874, Thermo Fisher). In particular, to eliminate lysosomal and peroxisomal contamination, cytosol components were centrifuged at 3000 × *g* for 15 min. Hsp90b protein was used as an indicator of cytosol contamination.

### 2.5 Mitochondrial ROS detection

MitoSOX Red (Invitrogen, Carlsbad, CA, USA) was used to measure mitochondrial superoxide production. In brief, the indicated cells were incubated with MitoSOX Red (5 μM) for 10 min at 37°C, followed by three times washes. The fluorescence intensity was examined using the CytoFLEX flow cytometry (Beckman Coulter) with the excitation wavelength at 488 nm and emission filter at 585/42 nm.

### 2.6 Mitochondrial membrane potential detection

Tetramethylrhodamine (TMRM; #T668, Invitrogen) was used to measure the mitochondrial membrane potential (MMP). Prepared cells were harvested and washed with Hanks solution. Cells were resuspended with 100 nM TMRM (diluted in cell growth medium) for 30 min at 37°C. The fluorescent signals were detected using the CytoFLEX LX flow cytometer (Beckman Coulter) with excitation at 561 nm and the emission filter at 585/42 nm.

### 2.7 Analysis of data from patients with gastric cancer

Two gastric cancer tissues were harvested in gastric cancer patients undergoing gastrectomy and received neoadjuvant therapy at the Zhongshan Hospital, Fudan University. The Research Ethics Committee of Zhongshan Hospital approved the clinical samples (No. B2017‐180R). Informed consent was obtained from participants included in the study.

The overall survival curves in three different types of gastric cancer (Lauren classifications) were generated using the online Kaplan–Meier plotter tool for gastric cancer.^17^ The analysis was based on the GEO series (GSE14210, GSE15459, GSE22377, GSE29272, GSE51105).

### 2.8 In vivo studies

Animals were housed in the the Animal Core Facility of Shanghai Institute of Biochemistry and Cell Biology. The IACUC committee approved the experimental procedures in the present study. A total of 1 × 10^6^ HGC27 *CCNC* ^–/–^ + *CCNC* ^WT^ or *CCNC* ^–/–^ + *CCNC* ^3ktoR^ cells were prepared in 100 μl medium and were injected into 4‐week‐old female nude mice subcutaneously (group 1, *CCNC* ^–/–^ + *CCNC* ^WT^ + saline; group 2, *CCNC* ^–/–^ + *CCNC* ^WT^ + cisplatin; group 3, *CCNC* ^–/–^ + *CCNC* ^3ktoR^ + saline; group 4, *CCNC* ^–/–^ + *CCNC* ^3ktoR^ + cisplatin; *n* = 3/group). When the average tumour size reached around 100 mm^3^, cisplatin was administered via intraperitoneal injection at a dose of 4 mg/kg every 4 days. Tumour volume was measured and calculated based on the equation: *V* = (length × width^2^)/2. All mice were sacrificed on the 40th day, and the tumours were dissected for TUNEL staining.

### 2.9 Statistical analyses

Data were analysed and plotted using GraphPad Prism‐7 (GraphPad Software). Statistical significances were performed by Student's two‐tailed paired or unpaired *t*‐tests. *p*‐Values <.05 were considered statistically significant (*p < .05, **p < .01). The results are presented as means ± SD. At least three independent replicates of experiments were conducted.

## 3 RESULTS

### 3.1 Cyclin C plays a vital role in cisplatin‐induced cell apoptosis via modulating mitochondrial stability in gastric cancer cells

**FIGURE 1:**
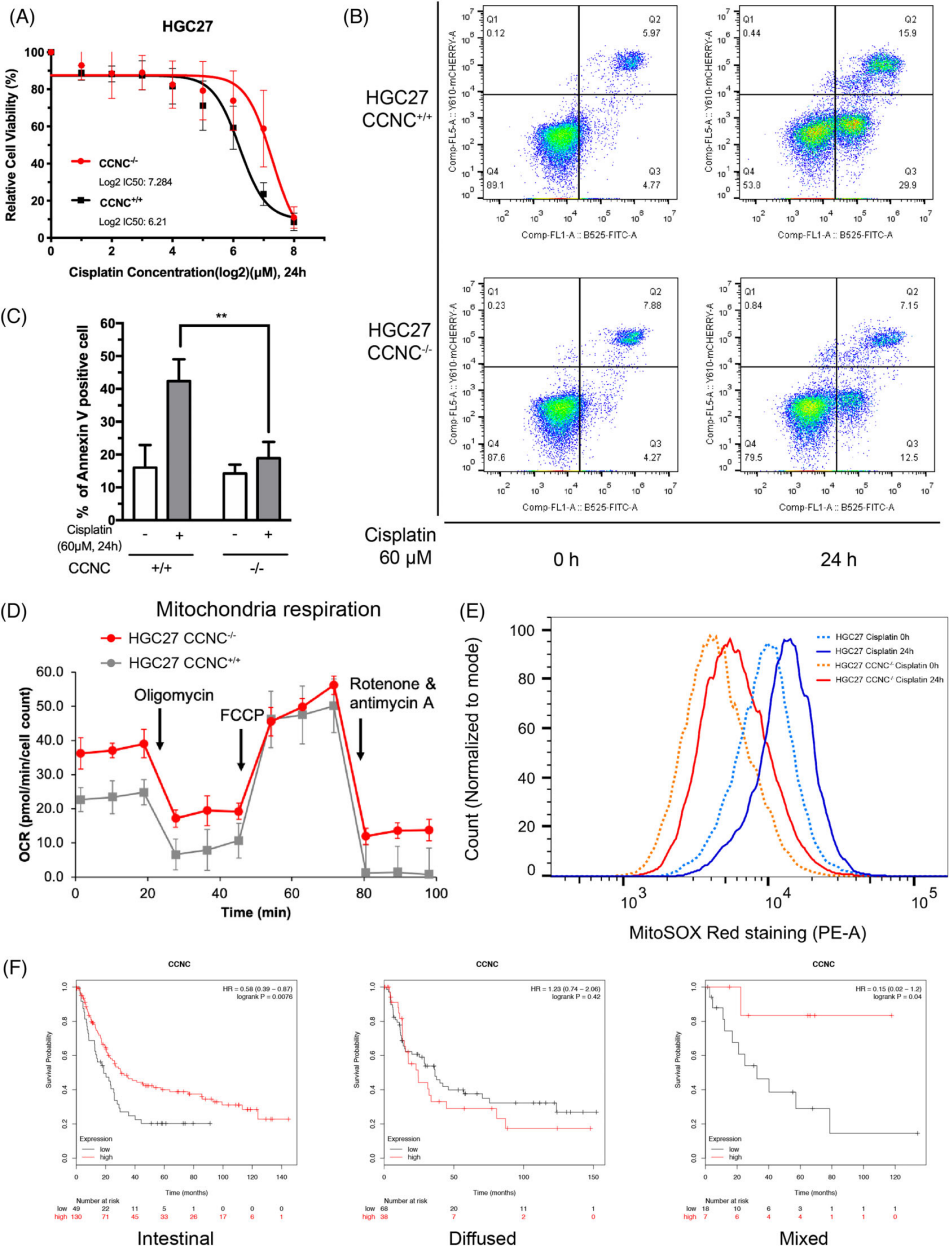
Cyclin C is essential for cisplatin‐induced cell apoptosis. (A) WT HGC27 cells and *CCNC* ^–/–^ HGC27 cells were treated with different doses of cisplatin for 24 h, and then cell viability for both groups was measured by adding CCK‐8 reagent and detecting the absorbance at 450 nm. And the half‐maximal inhibitory concentrations (IC_50_) were calculated in each group (*n *= 3, Log2 IC_50_: *CCNC* ^–/–^: 7.284, wildtype: 6.21). (B) The representative images of flow cytometry analysis of cell apoptosis in HGC27 and HGC27 *CCNC* ^–/–^ cells with or without 60 μM cisplatin treatment. The lower right quadrant (Annexin V+/PI−) identifies the early apoptotic cells, and the upper right quadrant (Annexin V+/PI−) identifies the late apoptotic cells. (C) Quantitative analysis of apoptotic cells (Annexin V+) detected by flow cytometry in (B) (*n *= 3, **p < .01, *p = *.0038, *CCNC* ^+/+^ vs. *CCNC* ^–/–^: 42.37 ± 6.652 vs. 18.88 ± 4.975, two‐tailed paired *t*‐test). (D) Seahorse measurement of oxygen consumption rate (OCR) in HGC27 and HGC27 *CCNC* ^–/–^ cells at different time points. (E) Mitochondrial ROS staining (MitoSOX Red) was detected in HGC27 and HGC27 *CCNC* ^–/–^ cells before (dot) and after (solid) cisplatin treatment (60 μM, 1 h). (F) Kaplan–Meier overall survival curve for gastric cancer patients, intestinal type (left), diffused type (middle) and mixed type (right) (red: high level of *CCNC* expression; black: low level of *CCNC* expression. Intestinal type: *p = *.0076, diffuse type: *p = *.42, mixed type: *p = *.04). All experiments were repeated three times independently. Error bars, SD of the mean and statistical comparisons were performed using Student's *t*‐tests

It has been reported that cyclin C participates in stress‐induced mitochondrial fission and apoptosis in mammalian cells.^8^, ^9^, ^18^, ^19^ Compared with control HGC27 cells, *CCNC* ^–/–^ HGC27 cells exhibited a higher IC_50_ when exposed to cisplatin in cell viability tests (Log2 IC_50_: control vs. CCNC^–/–^ 6.21: 7.284, Figure 1A), indicating that the depleting *CCNC* induces cell resistance to cisplatin. Flow cytometry assays reported that control HGC27 cells and *CCNC* ^–/–^ HGC27 cells had comparable annexin V signals under the basal condition. Cisplatin stimulation induced a significantly lower level of annexin V signals in CCNC^–/–^ cells compared with *CCNC* ^+/+^ HGC27 (*CCNC* ^+/+^ vs. *CCNC* ^–/–^: 42.37 ± 6.652 vs. 18.88 ± 4.975; *p* = .0038, Figure 1B,C). Seahorse influx analysis revealed that *CCNC* ^–/–^ cells had enhanced levels of mitochondrial respiration (Figure 1D) and lower levels of mitochondria‐derived ROS when stimulated with cisplatin (Figure 1E) in respect to oxygen consumption rate (OCR). The observation suggested that *CCNC* depletion in HGC27 cells retains mitochondrial function under cisplatin stimulation and protects cells through restraining the mitochondria‐derived ROS production.

Gastric cancer patients were classified into intestinal, diffused and mixed types according to Lauren classification.^20^ By using online Kaplan–Meier Plotter tool,^17^ Kaplan–Meier overall survival curve showed that gastric cancer patients, including intestinal type and mixed type, with higher expression of *CCNC* had a better survival rate (intestinal type: *p* = .0076; mixed type: *p* = .04; Figure 1F) compared with those with lower expressions of *CCNC*. However, a correlation was not found between *CCNC* levels and survival rate in patients with the diffused type (*p = *.42; Figure 1F). We also investigated the intestinal type and diffused type in TCGA database (mixed type cannot be found) and found the results were consistent with the GEO series. Specifically, in intestinal type, there was better survival rate in the high‐expression group (*p *= .048, Figure S1A), whereas in the diffused type, there was no significant difference in the survical rate between the high and low expression levels (*p *= .371, Figure S1B). These data indicated that the presence of cyclin C protein is crucial for cisplatin‐induced apoptosis in gastric cancer cells and its expression level is in positive correlation with intestinal type and mixed type of gastric cancer patients’ survival.

### 3.2 Tumour suppressor E3 ligase HACE1 targets cyclin C

**FIGURE 2:**
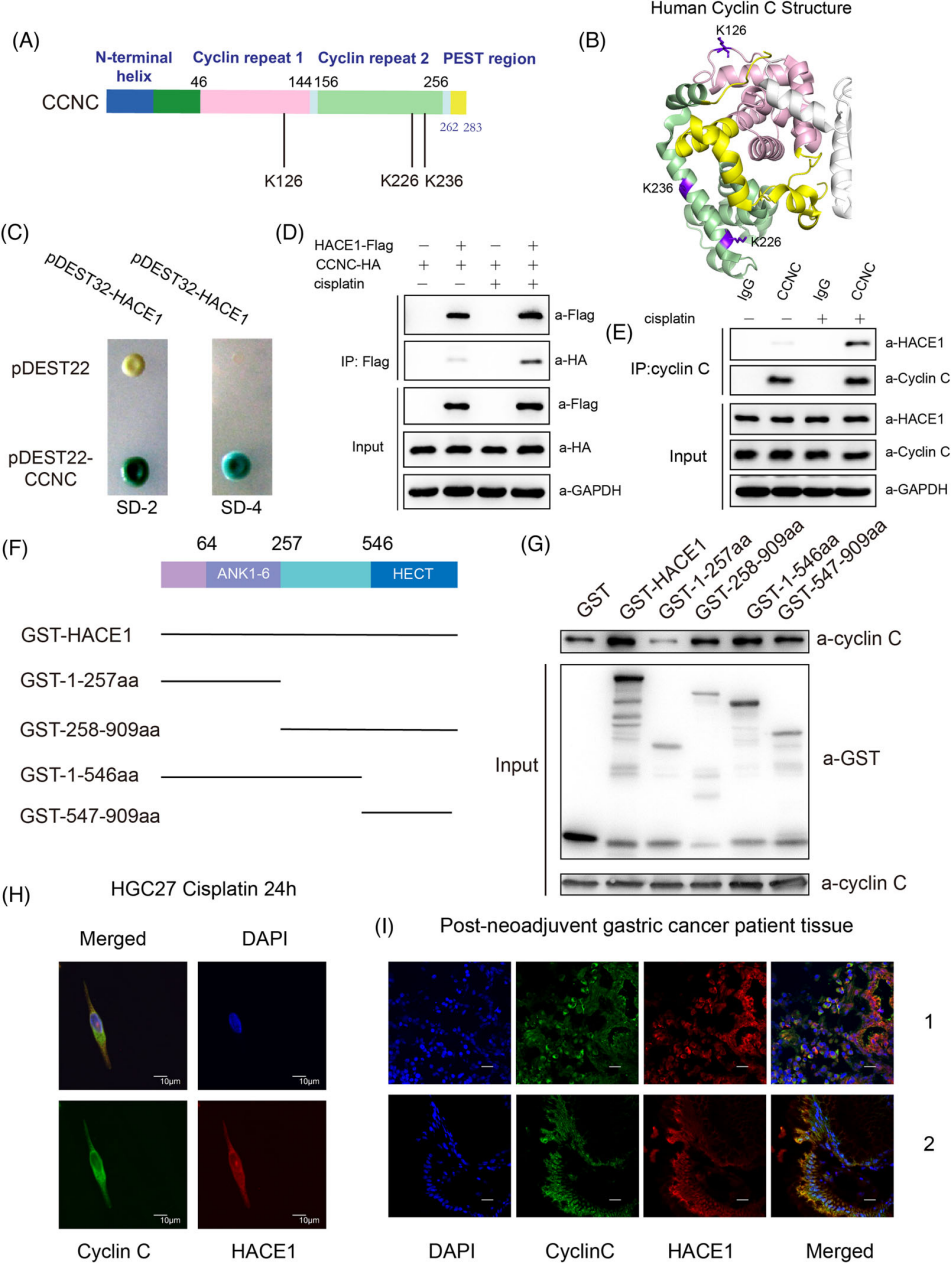
Cyclin C interacts with E3 Ub ligase HACE1 in vitro and in vivo. (A) Schematic representation of the domain architecture of cyclin C. Cyclin repeat 1: aa 46–144; cyclin repeat 2: aa 156–256. Major ubiquitylation sites: K126, K226 and K236. (B) The crystal structure of human cyclin C showing the three major ubiquitylation sites in purple (from the Protein Data Bank, PDB ID: 3RGF). Pink: cyclin repeat 1; green: cyclin repeat 2; white: part of CDK8. (C) Human cyclin C interacts with tumour suppressor HACE1 in the yeast two‐hybrid (Y2H) system. SD‐2, deficient in Leu and Trp; SD‐4, deficient in Leu, Trp, His and Ura. (D) The co‐immunoprecipitation assay that pulled down Flag‐tagged HACE1 and immunoblotted HA‐*CCNC* in HEK‐293FT cells without (left pannel) or with 10 μM cisplatin treatment (right pannel). (E) Endogenous co‐immunoprecipitation assay was performed by pulling down cyclin C using cyclin C‐specific antibody and immunoblotting HACE1 in HEK‐293FT cells without or with 10 μM cisplatin treatment. (F) Schematic diagram of HACE1 and its recombinant GST‐fused truncations. (G) GST pull‐down assays were performed by incubating different GST‐fused HACE1 recombinant proteins with MBP‐His‐tagged cyclin C, and then pulling down GST and immunoblotting cyclin C. (H) Immunofluorescence showing colocalisation of cyclin C (green) and HACE1 (red) in HGC27 cells stimulated with cisplatin for 24 h (scale bar: 10 μm). (I) Immunofluorescence showing colocalisation of cyclin C (green) and HACE1 (red) in tumour tissues from two gastric cancer patients receiving neoadjuvant therapy (scale bar: 20 μm). All experiments were repeated three times independently

As ubiquitylation is an important modification to proteins function, yeast two‐hybrid (Y2H) screening was performed to capture potential proteins that interact with cyclin C. Cyclin C contains two cyclin boxes, the N‐terminal helix and the PEST region (Figure 2A,B; Protein Data Bank, PDB ID: 3RGF). Colony formation on yeast with SD‐Leu‐Trp‐His‐Ura (SD‐4) selection media and plate assays for β‐galactosidase activity showed that HACE1 and cyclin C interacted (Figure 2C).

HACE1 is reported to enhance autophagy protein by ubiquitylation of optineurin protein.^11^, ^14^, ^15^ A co‐immunoprecipitation assay in both ectopically and endogenously expressing systems showed that cyclin C and HACE1 formed a complex in HEK‐293FT cells in response to cisplatin, which was barely observed in the absence of cisplatin (Figure 2D,E). We further investigated the influence of cisplatin on cyclin C translocation, which is discussed in detail in the following sections. Such kind of translocation is essential to facilitate the interaction between cyclin C and HACE1, as HACE1 is a cytoplasmic protein, whereas cyclin C exists in the nucleus under healthy condition and translocases to mitochondria when stimulated by H_2_O_2_ or cisplatin.^7^ In all, the data suggested that HACE1 has direct interaction with cyclin C in response to cisplatin in vivo.

Protein translocation is essential for the interaction between cyclin C and HACE1, as HACE1 is a cytoplasmic protein and cyclin C exists in the nucleus under the quiescent condition. To confirm the interaction of HACE1 and cyclin C, recombinant glutathione (GST)‐tagged HACE1 and its truncations were incubated with MBP‐His‐tagged cyclin C (Figure 2F). In GST pull‐down assays, truncated HACE1 at amino acids 1–257, but not at amino acids 258–909, 1–546 or 547–909, failed to form a stable complex with cyclin C (Figure 2G), suggesting the interaction between HACE1 and cyclin C has less relation to the ANK1–6 domain.

The immunofluorescence staining revealed that fluorescent signals of cyclin C and HACE1 were colocalised in the cytoplasm of cultured HGC27 challenged with cisplatin (10 μM) (Figure 2H) and in the samples from two gastric cancer patients who received neoadjuvant therapy (Figure 2I).

### 3.3 HACE1 ubiquitylates cyclin C with non‐proteolytic Ub lysine linkages

**FIGURE 3:**
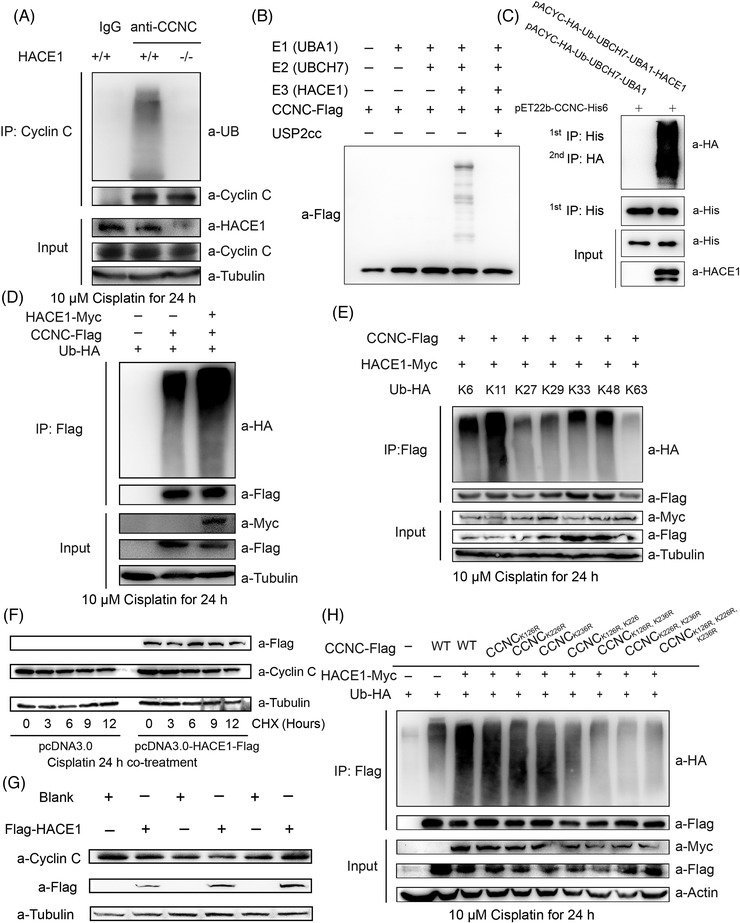
Cyclin C is ubiquitylated by HACE1 in non‐proteolytic linkages on lysine residues Lys126, Lys226 and Lys236. (A) Endogenous ubiquitylation assay was perfomed in HEK‐293FT cells after 10 μM cisplatin stimulation for 24 h. Cyclin C is immunoprecipitated from HEK‐293FT cells using anti‐cyclin C in modified RIPA buffer, followed by immunoblotting with anti‐Ub. (B) Human HACE1 ubiquitylated cyclin C in vitro. An in vitro ubiquitylation assay was carried out using cyclin C, UBA1 as E1, UbcH7 as E2 and HACE1, together with the indicated components USP2cc. (C) HACE1 ubiquitylated cyclin C in a reconstructed *E. coli* ubiquitylation system. (D) HACE1 enhanced cyclin C ubiquitylation in intact cells after cisplatin stimulation. HEK‐293FT cells were co‐transfected with Ub‐HA, *CCNC*‐Flag and HACE1‐Myc plasmids. Ubiquitylation‐conjugated cyclin C proteins were enriched using an anti‐Flag antibody and subjected to immune‐blotting using an anti‐HA antibody. (E) HACE1 ubiquitylated cyclin C with Lys11 ubiquitin linkages after 10 μM cisplatin treatment for 24 h. HEK‐293FT cells expressing HACE1‐Myc and HA‐Ub (Lys6, Lys11, Lys27, Lys29, Lys33, Lys48 or Lys63 only) as indicated. (F) HACE1‐mediated cyclin C ubiquitylation did not affect its protein stability. HACE1^–/–^ HEK‐293FT cells reintroduced ectoptic Flag‐HACE1, then cyclin C protein levels were detected by Western blot at different time points after cycloheximide treatment. (G) Transient overexpression of HACE1‐Flag does not alter cyclin C protein levels. Flag‐tagged HACE1 or Flag‐tagged vehicle (blank) were introduced into HEK‐293FT cells, and cyclin C protein levels were detected by immunoblotting. (H) Lys126, Lys226 and Lys236 are the major sites for HACE1‐mediated ubiquitylation of cyclin C. HEK‐293FT cells were transfected with Myc‐HACE1, HA‐ub and Flag‐tagged wild‐type cyclin C or its different mutants bearing single to multiple Lys‐to‐Arg substitutions at potential ubiquitylation sites. Then we pulled down flag‐tagged cyclin C and immunoblotting HA‐tagged ubiquitin. All experiments were repeated three times independently

The ubiquitylation on cyclin C was examined in the HEK‐293FT cells. In in vivo ubiquitylation assay, ubiquitin conjugation to cyclin C was observed in HEK‐293FT cells stimulated with cisplatin, but not in genetically modified *HACE1* ^–/–^ HEK‐293FT cells (Figure 3A). An in vitro ubiquitylation system composed of E1 Ub‐activating enzyme (UBA1), E2 Ub‐conjugating enzyme (UBCH7), E3 Ub ligase (HACE1), the substrate (cyclin C) and ubiquitin (Ub) was constructed. Consistently, cyclin C was notably ubiquitylated (Figure 3B), which was eliminated by USP2cc incubation, the catalytic core of human deubiquitylating enzyme USP2, suggesting the covalent conjugation of Ub in the process. Meanwhile, cyclin C was ubiquitylated when HACE1 was introduced in *E. coli*‐based ubiquitylation system (Figure 3C).^21^ Exogenous HACE1 plasmid also promoted the ubiquitylation of cyclin C in HEK‐293FT cells challenged with cisplatin (Figure 3D).

Seven lysine residues are reported in the ubiquitin molecule. Lysine (Lys) residues conjugate with methionine in an α‐amino group of the N‐terminal ubiquitin to form additional molecules, leading to regulating proteasomal degradation and mitophagy.^22^, ^23^, ^24^ To identify the specific Lys linkage of poly‐ubiquitin chains, multiple point mutations were designed, in which only one functional Lys residue was kept (Figure 3E). In the stimulation of cisplatin, ubiquitin molecules with Lys11 formed more poly‐ubiquitin chains on cyclin C than any other ubiquitin mutants, indicating that the poly‐ubiquitin chains on cyclin C were predominantly Lys11 ubiquitin linkages.

We next examined whether the ubiquitylation of cyclin C would affect the protein amount. Cycloheximide (CHX), an inhibitor of protein synthesis, did not change cyclin C degradation in HEK‐293FT cells stimulated with cisplatin (Figure 3F). Transient overexpressing HACE1 altered the cyclin C level (Figure 3G) in HEK‐293FT in the absence of CHX inhibition. These findings indicate that HACE1 ubiquitylates cyclin C via Lys11 linkages, but does not affect cyclin C degradation.

### 3.4 Lys126, Lys226 and Lys236 in cyclin C are the major sites for HACE1‐mediated ubiquitylation

As reported above (Figure 2A,B), three Lys residues, Lys126, Lys226 and Lys236 (K126, K226, K236), had potential to be ubiquitylated in vitro. To further identify ubiquitylation sites,^25^ cyclin C mutants carrying Lys‐to‐Arg (K‐to‐R) mutations were designed.

Single mutation of Lys126‐to‐Arg (K126R), Lys226‐to‐Arg (K226R) or Lys236‐to‐Arg (K236R) did not affect the conjugation, showing comparable ubiquitylation as wild‐type cyclin C (Figure 3H). Double mutation with K126R and K226R (*CCNC* ^K126R,K226R^) showed stronger ubiquitylate signals than the other double mutants (*CCNC* ^K126R, K236R^, *CCNC* ^K126R, K236R^), indicating that the ubiquitylation site of Lys236 is more important (Figure 3H). The mutation with all three sites (*CCNC* ^3KtoR^) abolished the ubiquitylation. These results suggested that there are multiple ubiquitylated sites.

### 3.5 Endogenous cyclin C translocates from nuclei to mitochondria under cisplatin stress and this translocation is influenced by HACE1‐mediated ubiquitylation

**FIGURE 4:**
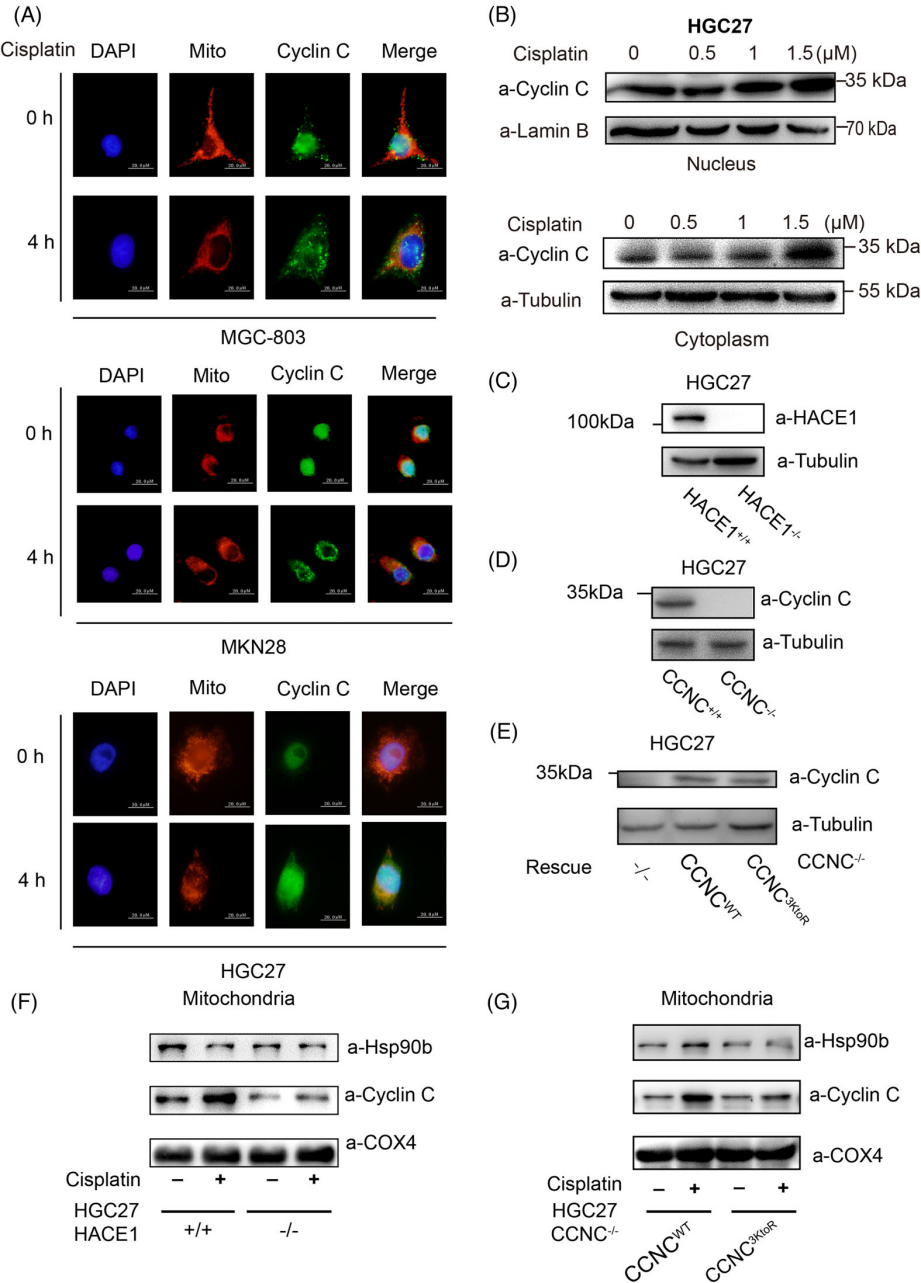
Ubiquitylation of cyclin C affects the amount of cyclin C on mitochondria under cisplatin stress. (A) Immunoflourescence was performed in in MGC‐803 (upper), MKN28 (middle), HGC27 (lower) gastric cancer cells to detect the cisplatin‐induced cyclin C (green) translocation from nucleus (indicated by 4′,6‐diamidino‐2‐phenylindole [DAPI], blue colour) to cytoplasm (indicated by mitochondria, red colour). (B) The amounts of cyclin C in the nucleus (up) and cytoplasm (down) were detected under cisplatin treatment with different drug concentrations in HGC27 cells. (C) The validation of the HGC27 *HACE1* ^–/–^ cell line using Western blot. (D) The validation of the HGC27 *CCNC* ^–/–^ cell line by Western blot. (E) The rescue of *CCNC* ^WT^ and *CCNC* ^3KtoR^ (with all three ubiquitylation sites mutated) in the HGC27 *CCNC* ^–/–^ cell line. (F) Cisplatin‐induced cyclin C translocation to mitochondria (lane 2 vs. lane 1; lane 3 vs. lane 4). Expression of cyclin C in mitochondria decreased in the HGC27 *HACE1* ^–/–^ cell line (lane 4 vs. lane 2). a‐Hsp90b serves as a mitochondrial marker, and COX4 serves as a reference protein. (G) Cisplatin‐induced cyclin C translocation to mitochondria (lane 2 vs. 1; lane 3 vs. 4). Expression of cyclin C in mitochondria decreased in HGC27 *CCNC* ^–/–^ + *CCNC* ^3KtoR^ cell lines (lane 4 vs. lane 2). All experiments were repeated three times independently

It is reported that cyclin C translocates to mitochondria when exposed to H_2_O_2_ or cisplatin, which is independent of CDK8.^9^ Indeed, cyclin C was found in mitochondria in MGC‐803, MKN28 and HGC27 gastric cancer cells stimulated with cisplatin (Figure 4A). The presence of cyclin C in cisplatin‐stimulated cells was increased in a dose‐ (Figure 4B) and time‐dependent manner (data not shown). These results indicated that cyclin C translocates from nuclei to mitochondria in response to cisplatin. Thus, further experiments were designed to study the protein ubiquitylation, as CDK8 is not involved in the process.

To study the role of HACE1‐catalysed ubiquitylation of cyclin C, a *HACE1*‐knockout HGC27 cell line (Figure 4C) and a *CCNC*‐knockout HGC27 cell line (Figure 4D) were established, respectively. There were no significant differences of the protein expressions between the wild‐type *CCNC* and *CCNC* ^3KtoR^ groups (Figure 4E). Cyclin C expression in the mitochondria was significantly reduced in *HACE1* ^–/–^ HGC27 cells, when compared with that of wild‐type cells stimulated with cisplatin (Figure 4F). Meanwhile, cyclin C failed to be ubiquitylated by HACE1, in *CCNC* ^3KtoR^‐rescued HGC27 cells, although there was a decrease in the translocation of cyclin C to mitochondria in response to cisplatin (Figure 4G). These results suggested that in response to cisplatin, the translocation of cyclin C from nuclei to mitochondria is regulated by HACE1‐mediated ubiquitylation.

### 3.6 Ubiquitylation of cyclin C is indispensable for increasing drug sensitivity to cisplatin in gastric cancer cells via modulating mitochondrial stability

**FIGURE 5:**
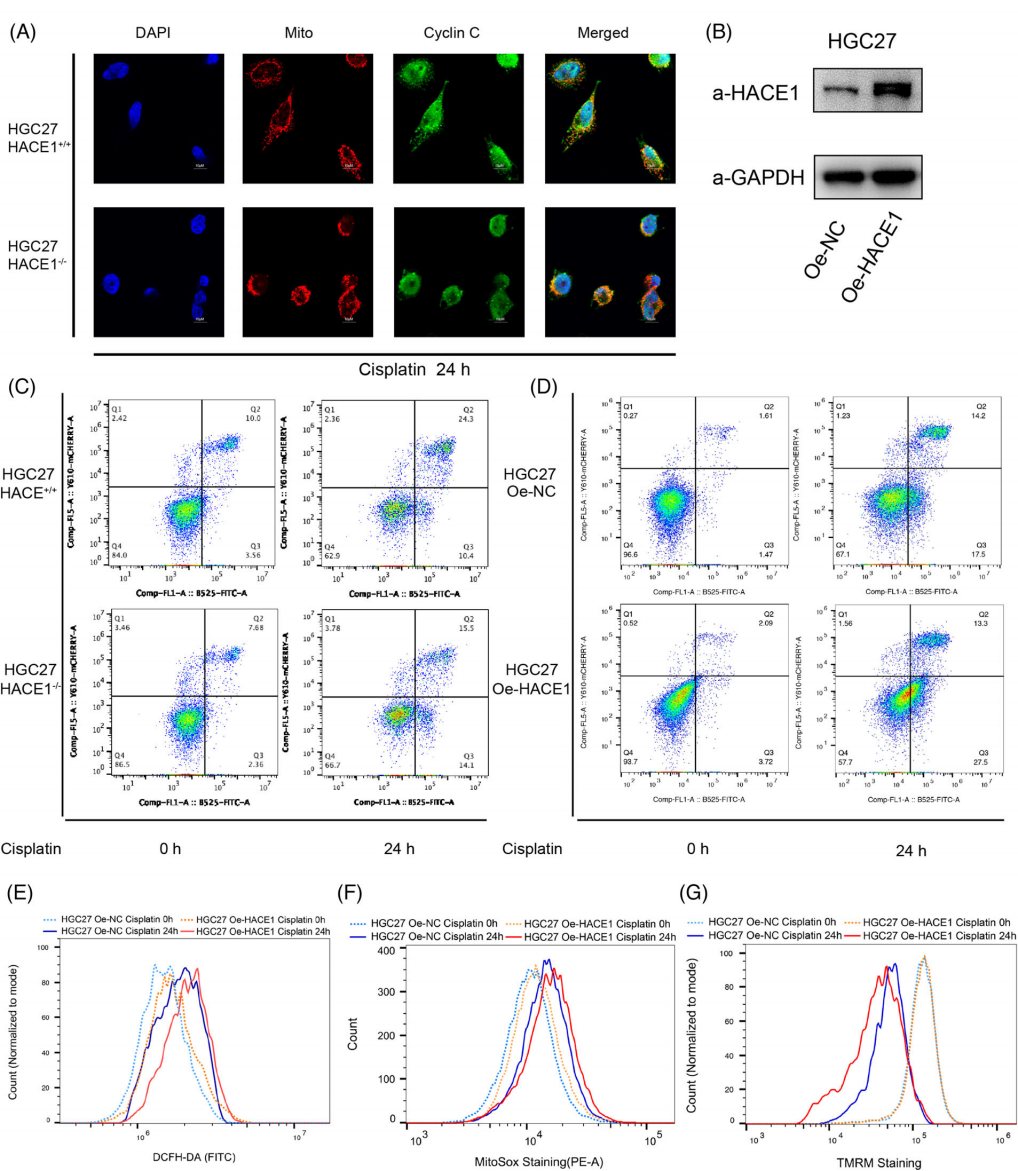
The depletion and overexpression of HACE1 influenced the cell apoptosis rate via mitochondrial dysfunction under the cisplatin stress. (A) Deletion of HACE1 does not affect the translocation process of cyclin C to mitochondria under cisplatin stress. Immunofluorescence was performed to detect the translocation of cyclin C (green) from nucleus (indicated by DAPI, blue) to mitochondira (red) in HGC27 cells with wild‐type HACE1 and with HACE1 knockout. (B) The validation of stably overexpressing ectopic HACE1 in HGC27 cells by Western blot. (C) The representative flow cytometry dot plot of the apoptotic assay in HGC27 and HGC27 *HACE1* ^–/–^ cells under basal and 60 μM cisplatin‐stimulated conditions. (D) The representative flow cytometry dot plot of the apoptotic assay in HGC27 Oe‐NC and HGC27 Oe‐HACE1 cells under basal and 60 μM cisplatin‐stimulated conditions. (E) Cellular ROS staining (DCFH‐DA) detected by flow cytometry in HGC27 Oe‐NC (blue) and HGC27 Oe‐HACE1 (red) cells under basal (dot) and cisplatin‐stimulated (solid) conditions. (F) Mitochondrial ROS staining (MitoSOX Red) detected by flow cytometry in HGC27 Oe‐NC (blue) and HGC27 Oe‐HACE1 (red) cells under basal (dot) and cisplatin‐stimulated (solid) conditions. (G) Mitochondrial membrane potential (MMP) staining (TMRM) detected by flow cytometry in HGC27 Oe‐NC (blue) and HGC27 Oe‐HACE1 (red) cells under basal (dot) and cisplatin‐stimulated (solid) conditions. All experiments were repeated three times independently

We next investigated whether ubiquitylation of cyclin C is essential for increasing drug sensitivity to cisplatin in gastric cancer cells via affecting mitochondrial stability. Initially, the drug sensitivity test of cisplatin did not show a significant difference between wild‐type and *HACE1* ^–/–^ HGC27 cells (Figure S2), which is probably due to a low expression of HACE1 as a tumour suppressor gene in cancer cells.^15^ Noteworthy, we intuitively confirmed that cyclin C translocated to mitochondria in *HACE1* ^–/–^ HGC27 cells challenged with cisplatin (Figure 5A). In the presence of cisplatin and Ac‐DEVD‐CHO, a caspase‐3 inhibitor, the presence of cyclin C in mitochondria was comparable in *HACE1* ^–/–^ HGC27 and control HGC27 cells, suggesting that caspase activity is not required in this process (Figure S3). It indicates that diminished levels of HACE1 affected the cyclin C translocation (Figures 4F and 5A).

As there was a decrease in the amount of cyclin C in mitochondria of *HACE1* ^–/–^ cells and *CCNC*‐depleted cells display reduced apoptosis activity, we then examined the apoptosis in *HACE1* ^–/–^ and Oe‐HACE1 cell lines (Figure 5B). The *HACE1* ^–/–^ cell line exhibited fewer annexin‐V signals than the wild‐type group in response to cisplatin (Figure 5C). Overexpressing HACE1 (Oe‐HACE1) significantly reduced annexin‐V signals (Figure 5D), increased oxidative stress (Figure 5E,F) and MMP (Figure 5G).

**FIGURE 6:**
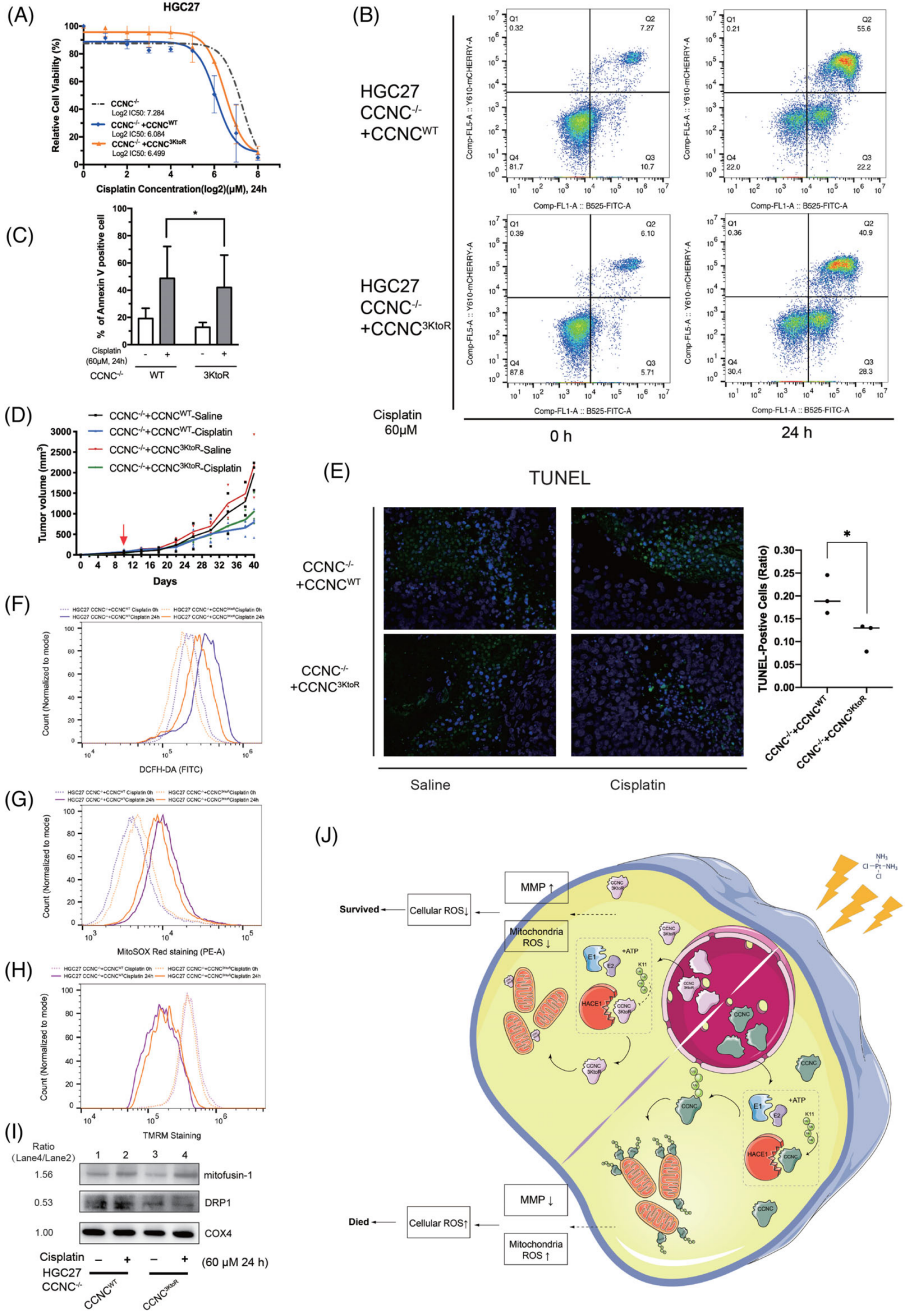
Ubiquitylation of cyclin C is indispensable for increasing cisplatin sensitivity in gastric cancer cells via modulating mitochondrial stability. (A) HGC27 *CCNC* ^3KtoR^ cells are resistant to cisplatin than wild‐type cells in the cell viability assay. *CCNC* ^–/–^ HGC27 cells with ectopic wild‐type cyclin C or mutated cyclin C were treated with diffrent doses of cisplatin for 24 h, and then cell viability for each group was measured by adding CCK‐8 reagent and detecting the absorbance at 450 nm. The half‐maximal inhibitory concentrations (IC_50_) were calculated in each group. (*n *= 3, Log2 IC_50_: *CCNC* ^–/–^ + *CCNC* ^3KtoR^: 6.499, *CCNC* ^–/–^ + *CCNC* ^WT^: 6.084). (B) The representative flow cytometry dot plot of the apoptotic assay in HGC27 *CCNC* ^–/–^ + *CCNC* ^3KtoR^ and *CCNC* ^–/–^ + *CCNC* ^WT^ cells under basal and cisplatin‐stimulated conditions. (C) Quantitative analysis of apoptotic cells (Annexin V+) detected by flow cytometry in (B). HGC27 *CCNC* ^3ktoR^ cells had less apoptotic cells than *CCNC* ^+/+^ cells in response to cisplatin under basal and conditions (*n* = 4, *p < .05, *p = *.0147, *CCNC* ^WT^ vs. *CCNC* ^3KtoR^: 48.77 ± 23.35 vs. 42.04 ± 23.76, two‐tailed, paired *t*‐test). (D) In vivo xenograft tumour growth experiments. Mice transplanted with HGC27 *CCNC* ^–/–^ + *CCNC* ^3KtoR^ and *CCNC* ^–/–^ + *CCNC* ^WT^ have comparable tumour sizes in response to cisplatin. (E) Representative images of TUNEL assay in tumour tissues and quantification result (*p *< .05). (F) Cellular ROS staining (DCFH‐DA) detected by flow cytometry in HGC27 *CCNC* ^–/–^ + *CCNC* ^3KtoR^ (orange) and *CCNC* ^–/–^ + *CCNC* ^WT^ (purple) cells under basal (dot) and cisplatin‐stimulated (solid) conditions. (G) Mitochondrial ROS staining (MitoSOX Red) detected by flow cytometry in HGC27 *CCNC* ^–/–^ + *CCNC* ^3KtoR^ (orange) and *CCNC* ^–/–^ + *CCNC* ^WT^ (purple) cells under basal (dot) and cisplatin‐stimulated (solid) conditions. (H) Measurement of mitochondrial membrane potential in HGC27 *CCNC* ^–/–^ + *CCNC* ^3KtoR^ (orange) and *CCNC* ^–/–^ + *CCNC* ^WT^ (purple) cells under basal (dot) and cisplatin‐stimulated (solid) conditions. (I) Representative immunoblotting of mitofusin‐1 and DRP1 in HGC27 *CCNC* ^–/–^ + *CCNC* ^3KtoR^ cells (lane 4 vs. lane 2). (J) Schematic diagram of this study. In brief, HACE1 ubiquitylates cyclin C via Lys11 Ub linkages in response to cisplatin treatment and promotes cyclin C translocation to mitochondria. HACE1‐induced ubiquitylation is blunted in cells with mutated cyclin C by 3KtoR, resulting in reduced mitochondria‐derived oxidative stress and enhanced resistance towards cisplatin. All experiments were repeated three times independently. Error bars, SD of the mean and statistical comparisons were performed using Student's *t*‐tests

Ubiquitylation sites point mutations of *CCNC* (*CCNC* ^3KtoR^) significantly shifted the cisplatin‐induced cell viability curve to the right (Log2 IC_50_: *CCNC*‐WT vs. *CCNC* ^3KtoR^ 6.084:6.499, Figure 6A). FACS revealed that in response to cisplatin, *CCNC* ^3KtoR^ cells had less annexin‐V signals than the *CCNC* ^WT^ group (*CCNC* ^WT^ vs. *CCNC* ^3KtoR^: 48.77 ± 23.35 vs. 42.04 ± 23.76; *p = *.0147, Figure 6B,C).

In the in vivo studies, xenograft tumour formation in nude mice were conducted in four groups: *CCNC* ^–/–^ + *CCNC* ^WT^ + saline, *CCNC* ^–/–^ + *CCNC* ^WT^ + cisplatin, *CCNC* ^–/–^ + *CCNC* ^3ktoR^ + saline and *CCNC* ^–/–^ + *CCNC* ^3ktoR^ + cisplatin. Cisplatin administration significantly reduced the tumour size in wild‐type mice. Mice with ubiquitylation sites point mutations of *CCNC* (*CCNC* ^3KtoR^) had comparable tumour size with wild‐type mice under the same condition (Figure 6D and Figure S4). However, counts of apoptosis cells were significantly reduced in *CCNC* ^3KtoR^, compared with wild‐type mice (Figure 6E).

Furthermore, we detected the cellular ROS production and mitochondrial function in these two groups. Flow cytometry reported that in response to cisplatin, the *CCNC* ^3KtoR^ group generated less cellular ROS and mitochondrial ROS, and maintained a stronger mitochondrial membrane integrity than the *CCNC* ^WT^ group (Figure 6F–H). Additionally, the *CCNC* ^3KtoR^ group also had improved mitochondrial respiration than *CCNC* ^WT^ in response to cisplatin (detected by OCR, Figure S4). In addition, given those previous findings have identified that mitochondrial fission and fusion are involved in mitochondrial stability and that cyclin C plays a vital role in stress‐induced mitochondrial fission,^9^, ^26^ mitofusin‐1, an indicator of mitochondrial fusion, and dynamin‐1‐like protein (DRP1), an indicator of mitochondrial fission, were examined. The results showed that the *CCNC* ^3KtoR^ group had reduced protein expression of DRP1 and increased expression of mitofusin‐1 than *CCNC* ^WT^ (Figure 6I). Therefore, the intact mitochondrial membrane integrity in the *CCNC* ^3KtoR^ group implied the necessity of HACE1‐mediated cyclin C ubiquitylation in increasing drug sensitivity to cisplatin via modulating mitochondrial stability in gastric cancer cells, and such kind of effect of ubiquitylated cyclin C may be exerted through regulating mitochondrial fusion and fission.

## 4 DISCUSSION

Cisplatin has been widely applied in chemotherapy for various types of cancers as it interferes with DNA replication, exerting lethal effects on fast proliferating cells. However, the emerging cisplatin resistance becomes a big issue for cancer treatment. Cisplatin‐associated resistance usually develops in tumour recurrence, attributed to increased DNA repair, altered cellular accumulation and increased drug inactivation.^27^, ^28^ In the present study, we elucidated a novel mechanism by which disorganisation of HACE1–cyclin C interaction drives gastric cancer cell resistance to cisplatin treatment by deregulating mitochondrial‐associated oxidative stress.

Cyclin C is a nuclear protein, while its nuclear–mitochondrial translocation, in response to oxidative stress, has been found in yeast^7^, ^29^, ^30^ and mouse embryonic fibroblast.^9^ Studies have reported that the cytoplasmic cyclin C interacts with mitochondrial division 1 protein, an adaptor linking dynamin 1 to its mitochondrial receptor fission 1,^7^ as well as recruiting Bax protein to mitochondrial outer memberane,^31^ which implicates an interplay of cyclin C in regulating mitochondrial function. In the present study, cisplatin‐induced cyclin C translocation was also observed in MGC803, MKN28 and HGC27 cells, three human gastric cancer cell lines, indicating that the nuclear–mitochondrial translocation of cyclin C is a general phenomenon in cisplatin stimulation.

HACE1 is an E3 ligase, which resides in cytoplasm and functions as a tumour suppressor in different cancers. We found that HACE1 and cyclin C formed a complex when cyclin C translocated into cytoplasm under cisplatin stimulation, suggesting that cyclin C is a potential ubuiquitylation substrate of HACE1. Such interaction can only take place when cisplatin is administrated. Under the treatment of cisplatin, cyclin C translocates to cytoplasm where it would be ubiquitylated by HACE1. Cisplatin could be crucial to this interaction also by inducing some posttranslational modifications to other proteins, for example, histones.^32^ Further studies are required to fully decode the role of cisplatin in such kind of interactions. In this research, we mainly investigated the ubiquitylation and its significance. Indeed, we found that HACE1 ubiquitylates cyclin C through linkages K11 at Lys126, Lys226 and Lys236 of cyclin C protein. Ubiquitylation, a posttranslational protein modification, affects protein function by regulating protein degradation, protein location and/or protein interactions.^33^ Then we were interested in exploring how the modificiation of cyclin C affects its function. Interestingly, we found that the K11‐ubiquitin chain modification did not change cyclin C protein stability, suggesting the K11 linkage ubiquitylation of cyclin C is non‐proteolytic. Usually, seven lysine residues and an N‐terminus of ubiquitin are served for protein ubiquitination, including K6, K11, K27, K29, K33, K48, K63 and M1.^24^ The role of K48 and K63 is well studied for facilitating or preventing proteasomal degradation of the substrate protein, respectively.^34^, ^35^, ^36^, ^37^, ^38^ K11‐linked chains are often accompanied by the K48 counterparts in regulating protein degradation involved in cell mitosis,^39^ as well as protein expressions of type 1 interferon^40^ and hypoxia‐inducible factors HIF1α and HIF2α.^41^ Although Lys11‐linked Ub chains have been regarded as proteasomal signals in cell cycles in APC/C signaling pathway,^42^ recent evidence has shown that homotypic K11 linkages do not direct the substrate for proteasomal degradation, instead, the heterotypic K11 linkages are efficient for targeting proteasomal degradation.^43^ Therefore, one explanation could be that the K11 linkage ubiquitylation of cyclin C is non‐proteolytic as K11 chains are homotypic. However, further study is needed to define the assembly pattern of this K11‐Ub chains. We consider it to be a novel and important supplement to the role of K11 linkages.

Then we found that HACE1‐mediated cyclin C ubiquitylation is crucial for cell viability. Inhibiting cyclin C ubiquitylation by mutating cyclin C ubiquitylation sites or by deleting HACE1 in HGC27 cells did not prevent cyclin C nuclear–mitochondrial translocation, but reduced mitohondiral‐derived ROS synthesis and mitigated cell apoptosis. It further suggests that mutation on the three residues could be the possible mechanism underlying cisplatin‐associated resistance in gastric cancer patients, yet requires further investigation.

Our discovery suggests that depletion of HACE1, which is prevalent in gastric cancer,^15^ is one of the possible mechanisms underlying cisplatin‐associated resistance in gastric cancer patients. However, previous studies from other research groups have revealed the involvement of HACE1 in reducing ROS synthesis by regulating RAC1‐dependent ROS synthesis and the nuclear factor erythroid 2‐related factor 2 (NRF2)‐associated oxidative stress response. By catalysing the ubiquitylation and degradation of NADPH‐bounded RAC1, HACE1 inhibits ROS generation from the NADPH oxidase complex.^44^ In mouse brain, HACE1 executes antioxidative activity by activating NRF2 transcription factor, which translocates to nucleus upon activation and enhances expression of antioxidant protein.^45^ Then knocking out HACE1 leads to increased oxidative stress in the brain. Another group found that HACE1 loss‐of‐function mutations reduced mitophagic flux and increased mitochondrial oxidative stress in human fibroblasts.^46^ While in human gastric cancer cell lines, we found that loss of HACE1 reduced oxidative stress and promoted cisplatin resistance by preventing cyclin C‐induced mitochondrial dysfunction and ROS synthesis. The controversial role of HACE1 in gastric cancer cells, fibroblasts and mouse brain suggests that HACE1's regulation of oxidative response exists in molecular background specificity. Comparing to noncancer cells like neurons and fibroblasts, cancer cells carry a unique feature of genomic instability in which the gene expression profile is unstable and dramatically changes to favour aggressive growth and avoid detrimental effect from therapeutic drugs. Hence, it is possible that in the altered molecular context in gastric cancer cells, HACE1‐Rac1‐NADPH functional pathway, HACE1‐NRF2‐antioxidant proteins pathways and the HACE1‐mitophagy pathway are perturbed, or other pathways may become dominant to prevent their unfavourable effect for cancer growth. However, the exact mechanism is unknown, and more efforts are required for future studies.

In this study, we find that loss of HACE1 inhibits ROS synthesis under cisplatin stress, which helps the gastric cancer cells survive cisplatin treatment. This observation indicates that HACE1 acts as a tumour suppressor in gastric cancer, which is consistent with previous findings in our lab and by others.^15^, ^47^, ^48^

Taken together, with cisplatin‐induced nuclear–mitochondrial translocation of cyclin C, its ubiquitylation by HACE1 increased mitochondrial fission and mitochondrial‐derived oxidative stress, leading to cell apoptosis (Figure 6J). Mutation on the ubiquitylation sites of cyclin C inhibits cisplatin‐induced cell death, shedding light on a better understanding of cisplatin‐associated resistance in gastric cancer patients. In addition, Stieg et al.^19^ reported that cyclin C directly modulates stress‐related genes transcription, including p53‐induced genes and those that are essential for autophagy. Thus, further studies may also investigate whether the ubiquitylation of cyclin C would affect the transcriptional function of cyclin C on stress‐related genes. Precision diagnosis and treatments regarding cyclin C ubiquitylation sites are requested in future clinical practice.

## CONFLICT OF INTEREST

The authors declare that they have no competing interests.

## Supporting information

Supporting Information
