# Supplementary material for: Ubiquitylation of cyclin C by HACE1 regulates cisplatin‐associated sensitivity in gastric cancer
Source: Clin Transl Med. 2022 Mar 28;12(3):e770. doi: 10.1002/ctm2.770 (PMC8958351; doi:10.1002/ctm2.770)
Supplement: Supplementary file 1 — Supporting Information [file CTM2-12-e770-s001.docx]

Supplementary Materials for

**Ubiquitylation of Cyclin C by HACE1** **regulates gastric cancer cell cisplatin sensitivity via modulating mitochondria stability**

**Running title:** *Ubiquitylation of Cyclin C by HACE1 in GC*

**Authors：**Hong-yue Jiang^1*^, Ying-ling Chen^1*^, Xing-xing Xu^2*^, Chuan-yin Li^2,3^, Yun Chen^1^, Dong-ping Li^1^, Xiao-qing Zeng^1^, Hong Gao^1,4^

**Affiliations:** ^1^Department of Gastroenterology and Hepatology, Zhongshan Hospital, Fudan University, Shanghai, China, 200032; ^2^State Key Laboratory of Molecular Biology, CAS Center for Excellence in Molecular Cell Science, Innovation Center for Cell Signaling Network; Shanghai Institute of Biochemistry and Cell Biology, Chinese Academy of Sciences, Shanghai, China, 200032; ^3^University of Chinese Academy of Sciences, China; ^4^ Evidence-based Medicine Center of Fudan University, Shanghai, China.

**Corresponding Author:**

Dr. Gao, Hong and Dr. Zeng, Xiao-qing

#180, Fenglin Road, Zhongshan Hospital, Fudan University, Shanghai, China PRC CN-200032

Phone number: +86-13611673691

Fax: +86-021-64432583

E-mail: [gao.hong@zs-hospital.sh.cn](mailto:gao.hong@zs-hospital.sh.cn) and zeng.xiaoqing@zs-hosoital.sh.cn

^*^ These authors contributed equally to this work

**Supplementary Materials and Methods**

## Yeast Two-Hybrid Screen

Yeast two-hybrid (Y2H) screening was used to determine the E3 Ub ligases that interact with cyclin C, as described previously.^1^

## Immunoprecipitation

The indicated cells with designated endogenous or exogenous protein expressions were lysed in IP buffer (50 mM Tris-Cl, pH 7.5, 150 mM NaCl, 1 mM EDTA, 1% NP-40, 10% glycerol) addition with protease inhibitor cocktail present (Bimake, Houston, TX, USA) and sonicated using the ultrasonic cell crusher noise isolating chamber (OURSultrasonic, Shenzhen, Guangdong, China). Immunoprecipitation was performed as previously described.^2^ The primary antibodies for immunoprecipitation were: normal rabbit IgG (sc-2729, Santa Cruz Biotechnology, Inc., Dallas, TX, USA), anti-FLAG®M2 affinity gel (A2220, Sigma-Aldrich, St Louis, MO, USA), cyclin C (A301-989A, Bethyl, Montgomery, TX, USA), HA (H6908, Sigma-Aldrich), His (66005-1-lg, Proteintech, Rosemont, IL, USA).

## Immunofluorescence Assay

The indicated cells were seeded on cover glasses (VWR, Radnor, PA, USA) and treated in the presence or absence of cisplatin with indicated time intervals in 24-well chambers before immunofluorescent staining. For Mitotracker (Molecular Probes, Eugene, OR, USA) staining, after cells were washed with PBS, 500 μl Mitotracker (100 nM) was added, followed by 30 min incubation in 37 ℃. Cells were fixed in 4% paraformaldehyde and permeabilized by 1‰ Triton X-100 (Diamond, Shanghai, China). Five percent BSA (Yeasen, Shanghai, China) was then used for blocking. Subsequently, cells were incubated with the primary antibody (cyclin C 1:200, HACE1 1:200, diluted in 1% BSA) at 4 °C overnight, and then the Alexa Fluor 488-conjugated goat-anti-rabbit antibody (Molecular Probes) and goat-anti-mouse-Cy3 (Jackson Immunoresearch, West Grove, PA, USA) secondary antibody at room temperature for1 h. The cover glass were mounted (DAPI Fluoromount-G, SouthernBiotech, Birmingham, AL, USA). Images were acquired on the BX51 fluorescence microscope with 100×oil (Olympus, Tokyo, Japan) and Olympus FV1200 confocal microscope with 60×oil (Olympus) according to the study design. For tissue immunofluorescent staining, tissues were circled with a PAP pen and blocked by goat serum (Boster, Wuhan, Hubei, China) for 30 min

## *In vivo* and *In vitro* Ubiquitylation Assays

For the *in vivo* ubiquitylation assay, endogenous or exogenous cyclin C was immunoprecipitated from cells treated with proteasome inhibitor Bortezomib (BTZ, VELCADE^®^, Takeda, Tokyo, Japan). *In vivo* ubiquitylation was performed as described previously.^1^

For the *in vitro* ubiquitylation assays, *E. coli* expressed human Ubiquitin (His6-Ub), Ub-activating enzyme E1 (His6-UBA1), Ub-conjugating enzyme E2 (His6-UbcH7), Ub ligase E3 (GST-HACE1), and cyclin C (cyclin C-Flag-His6) were purified. One hundred nanograms of UBA1 (E1, ubiquitin activating enzyme), 150 ng UbcH7 (E2, ubiquitin conjugating enzyme), 500 ng HACE1, 500 ng cyclin C and 200 ng Ub proteins were added to the ubiquitylation buffer, and the reaction was performed as described previously.^1^

To determine the type of ubiquitin linkages of the poly-Ub chains conjugated to cyclin C by HACE1, HEK-293FT cells with stably expressed Myc-tagged HACE1 were transiently transfected with pCDNA3.0-Flag-CCNC and equal amounts of pRK5-HA-Ub (UbK6, UbLYS11, UbK27, UbK29, UbK33, UbK48, or UbK63) plasmids, as described previously.^1^ Cells were harvested 48 h after transfection and sonicated. The cell lysates were centrifuged at 22500 *g* and 4 ℃ for 15 min. One-tenth of the supernatants were loaded to immunoblotting to confirm the equal amount of input, whereas the remaining materials were incubated with an anti-Flag affinity gel for 6 h at 4 ℃. The recovered beads were then washed three times, followed by immunoblotting analysis using anti-HA and anti-Flag antibodies.

## *E. coli* ubiquitylation system reconstitution

The *E. coli* ubiquitylation system reconstitution was performed as described previously.^2^ In brief, HACE1 was inserted into the second multiple cloning site of the pACYCDuet-1 vector (Novagen^®^, Merck, Darmstadt, Germany), to generate the plasmid pACYC-HA-UB-UBCH7-UBA1-HACE1. *E. coli* BL21 competent cells were co-transformed with pACYC-HA-UB-UBCH7-UBA1/pACYC-HA-UB-UBCH7-UBA1-HACE1 and pET22b-CCNC-His6 plasmids by electroporation and selected with chloramphenicol and ampicillin antibiotics. After proper culturing, the *E. coli* cells were then sonicated and centrifuged. Cyclin C protein was purified from the supernatant using Ni-NTA agarose beads and the ubiquitylation level was examined by the immunoblotting assay.

## Mass Spectroscopy And Mutagenesis Analyses

MS and mutagenesis analyses were performed to identify the amino acid sites of cyclin C that HACE1 conjugates poly-Ub chains to. Samples were generated via the same *in vitro* ubiquitylation protocols mentioned above. The handling of the sample was as described before.^3^ Desalting and concentrating of the samples was performed with StageTip.^4^ Finally, the eluted peptides were submitted to MS analysis.

## Plasmid construction, virus infection and establishment of stable cell lines

Plasmids used in this study are listed in the Supplementary Information (Supplementary Table S1). Briefly, restriction enzyme digestion and ligation reactions (New England Biolabs, Beverly, MA, USA). Briefly, restriction enzyme digestion and ligation reactions (New England Biolabs) were performed using traditional cloning methods. Point mutations were introduced by site-directed mutagenesis using KOD-Plus-Neo (Toyobo, Kita-ku, Osaka, Japan) and Dpn I (ER1701, Thermo Fisher).^5^ Lentivirus was produced from HEK-293FT cells with the lentiviral packaging mix and the expression plasmids. Selected culture medium with puromycin (2 μg/mL, Sigma-Aldrich) was subsequently used to construct stable cell lines and validation was performed by immunoblotting.

## Seahorse assay

The Seahorse XFe Analyzer (Agilent, Santa Clara, CA, USA) was used to perform the cell mitostress test assay according to the manufacturer’s instructions. HGC-27 cells (2 × 10^4^) were seeded on the Seahorse XF24 V7 PS Cell Culture Microplates (No.100777-004, Agilent) and cultured overnight in normal growth medium. The sensor cartridge was hydrated overnight. Seahorse assay medium (10 mM glucose, 1 mM sodium pyruvate, 2mM glutamine added when needed, pH 7.4) was prepared and stored at 4 ℃. Cells were carefully washed with assay medium the following day and incubated in assay medium at 37 °C for 1 hour. The sensor cartridge was loaded with the compounds (1 μM oligomycin, 0.5 μM FCCP, 1 μM rotenone, and antimycin A, diluted in assay medium) (XF Cell Mito Stress Test Kit, Agilent) into each reagent port. The plate, together with the cartridge, was placed into the XFe Analyzer to run the experiment. After the Seahorse assay experiment, each well of the plate was stained with Hoechst and counted using EnSight (PerkinElmer, Waltham, MA, USA) to normalize the cell number. Data were analyzed by using the Seahorse Wave Desktop (Agilent).

## Cell viability assays

HGC27 cells were seeded in 96-well plates at 8000 cells/well overnight. Nine different concentrations of cisplatin (0, 2, 4, 8, 16, 32, 64, 128 and 256 μM) were then administrated to cells in a logarithmic dose pattern with three replicates for 24 h. CCK-8 (C0042, Beyotime Biotechnology) was used to detect cell viability at 450 nm/600 nm after incubation for 1 hour according to the manufacturer's instruction. Three independent repeats were collected. The dose-inhibition curve and IC_50_ were calculated by using GraphPad Prism-7 software (GraphPad Software, La Jolla, CA, USA).

## Stress and Apoptosis assays

HGC27 cells were seeded in 12-well plates at a density of 0.5 × 10^5^ cells/well overnight before cisplatin treatment. Cisplatin (S116613, Selleckchem, Houston, TX, USA) was diluted from a stock solution (20 mM) to a working solution (60 μM) in the normal culture medium. After cisplatin treatment for 24 h, cells were harvested for apoptosis detection. Apoptosis was investigated using the annexin V-fluorescein isothiocyanate (FITC) Apoptosis Detection kit (AD10, DOJINDO, Kumamoto, Japan) in accordance with the manufacturer's instructions and examined on a CytoFLEX LX flow cytometer (Beckman Coulter, Atlanta, Georgia, USA). At least three independent experiments were performed.

## Nuclear and cytoplasmic extract preparation

The nuclear and cytoplasmic extract preparation required buffer A (10mM HEPES, 1.5mM MgCl_2_, 10 mM KCl, 0.5 mM DTT added when used, pH 7.9) and buffer A' (buffer A plus 0.2% NP-40 and 1× protease inhibitor cocktail). Buffer A was prepared for multiple uses as stock (without DTT), and buffer A' was prepared when required. Cells were seeded in 6/12-wells and treated with or without cisplatin. Harvested cells were washed with PBS and centrifuged. Each cell pellet sample was resuspended with 500 μl buffer A and incubated on ice for 10 min. Cells were then centrifuged at 2500 *g* for 2 min, and the supernatants were removed. Cell pellets were then resuspended with 100 μl buffer A' and centrifuged immediately at 5000 *g* for 1 min. The supernatants were then loaded with 6× SDS as cytoplasmic proteins, whereas the pellets were dissolved in 1× SDS as nuclear proteins. The protein samples were analyzed by immunoblotting.

## Cellular reactive oxygen species (ROS) levels

The amount of cellular ROS was measured with the Reactive Oxygen Species Assay Kit (S0033, Beyotime, Nanjing, China). Briefly, cells were treated with or without cisplatin (60 μM) for 24 h. Cells were harvested and incubated with DCFH-DA (10μM) diluted in the serum-free medium according to the manufacturer’s instruction at 37 °C for 20 min. The DCF fluorescence distribution was examined by CytoFLEX LX(Beckman Coulter) with the excitation wavelength at 488nm and emission at 525/40nm.


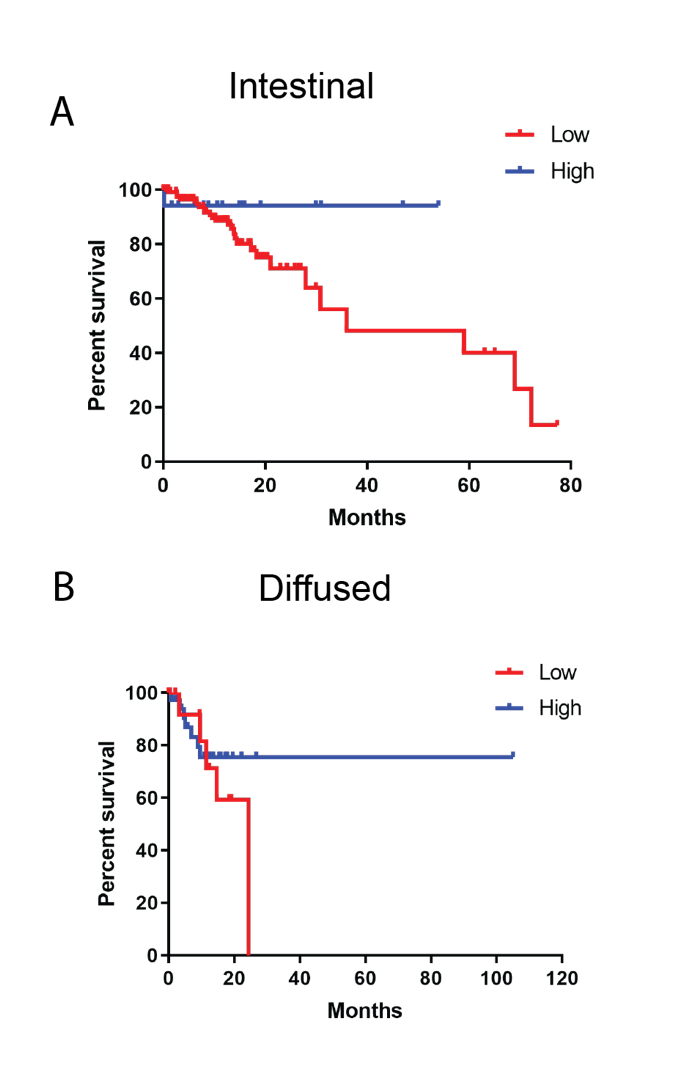


**Supplementary Fig S1.** **Kaplan-Meier overall survival curve in two different cyclin C expression level group (low and high) for gastric cancer patients in TCGA database.** A. intestinal type, *p*=0.048, B. diffused type, *p*=0.371

**
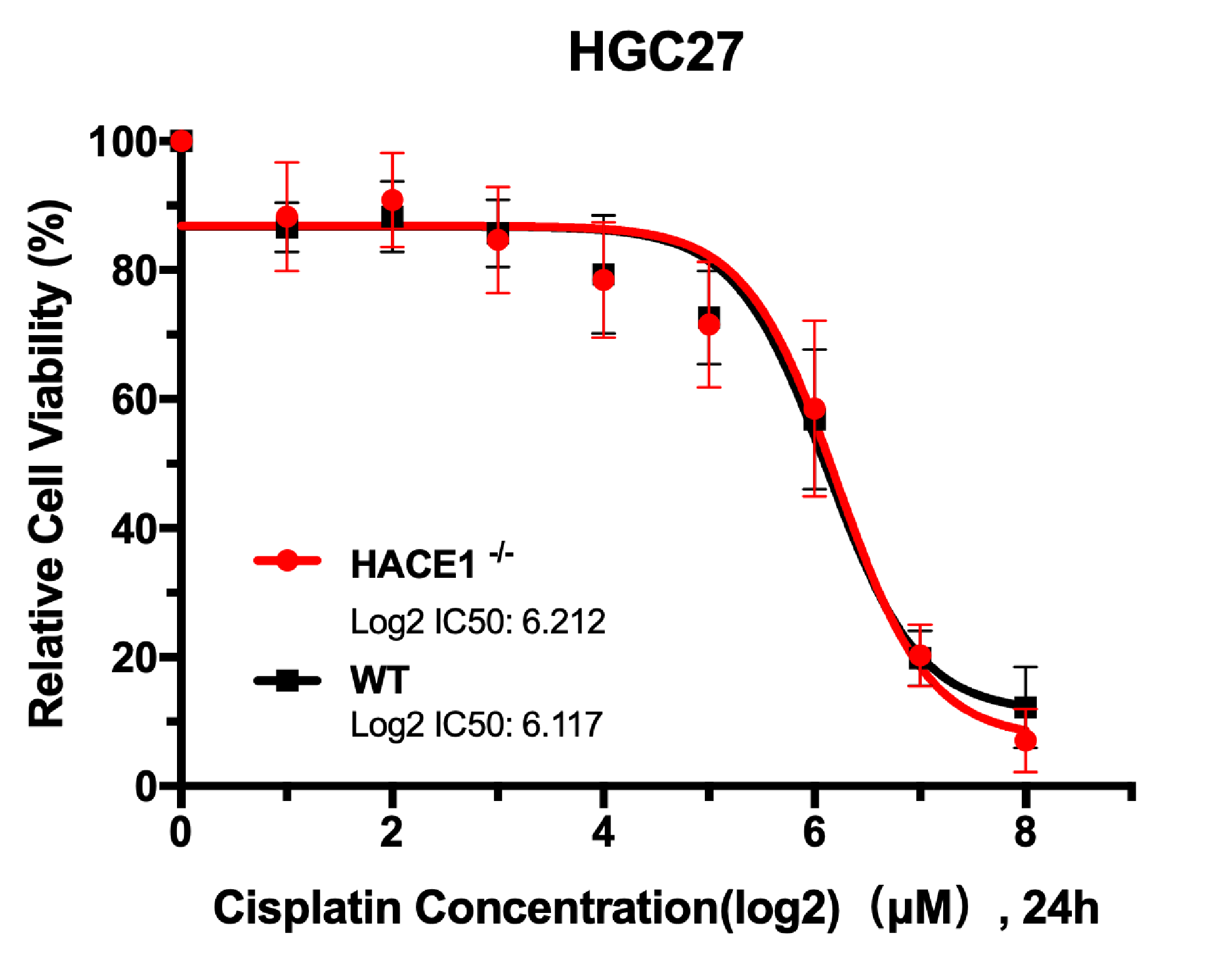
**

**Supplementary Fig. S2. Relative cell viability of HGC27 and HGC27 HACE1^–/–^**

Relative cell viability of HGC27 and HGC27 HACE1^–/–^ under a series of cisplatin concentrations (0, 2, 4, 8, 16, 32, 64, 128, 256 μM).


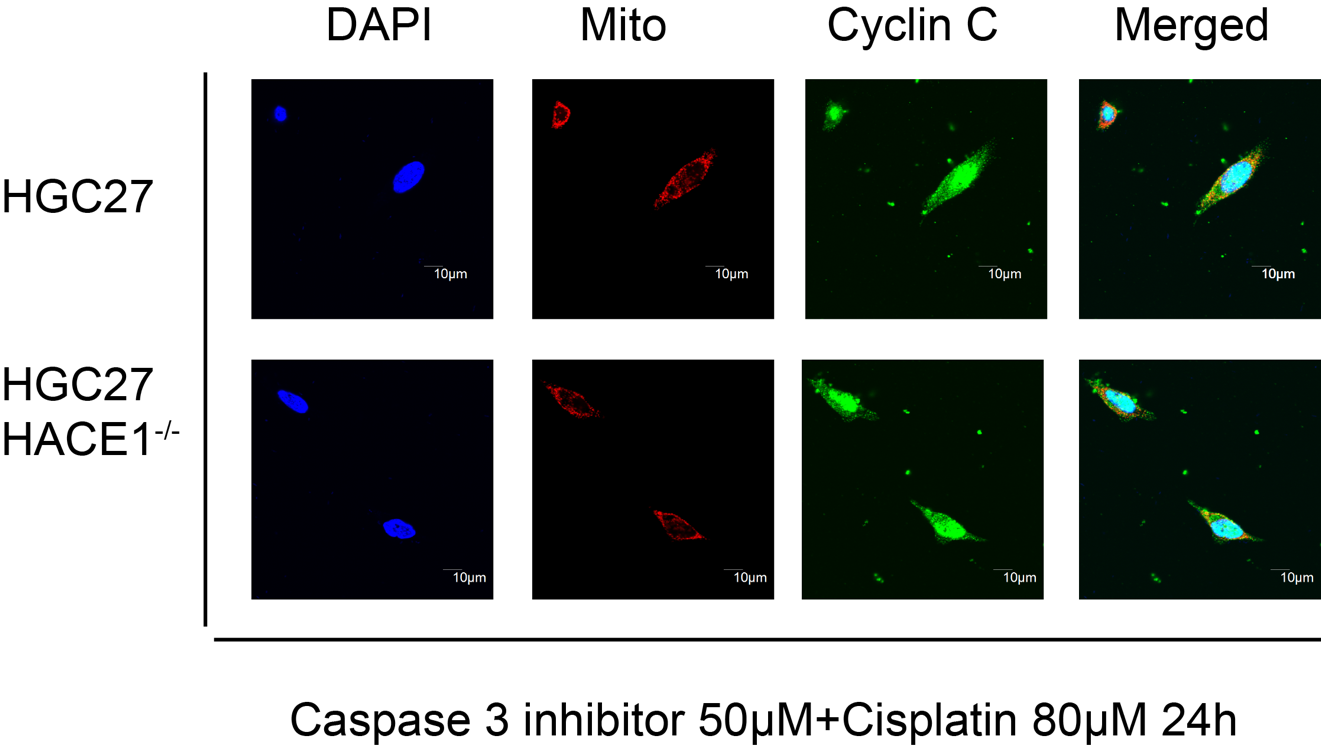


**Supplementary Fig. S3. Immunofluorescence staining of mitochondria and cyclin C after co-administration of caspase 3 inhibitor with cisplatin.**

HGC27 and HGC27 HACE1^–/–^ culture was co-treated with the caspase inhibitor Ac-DEVD-CHO (50 μM) for 1 h and then cisplatin (80 μM) for 24 h.


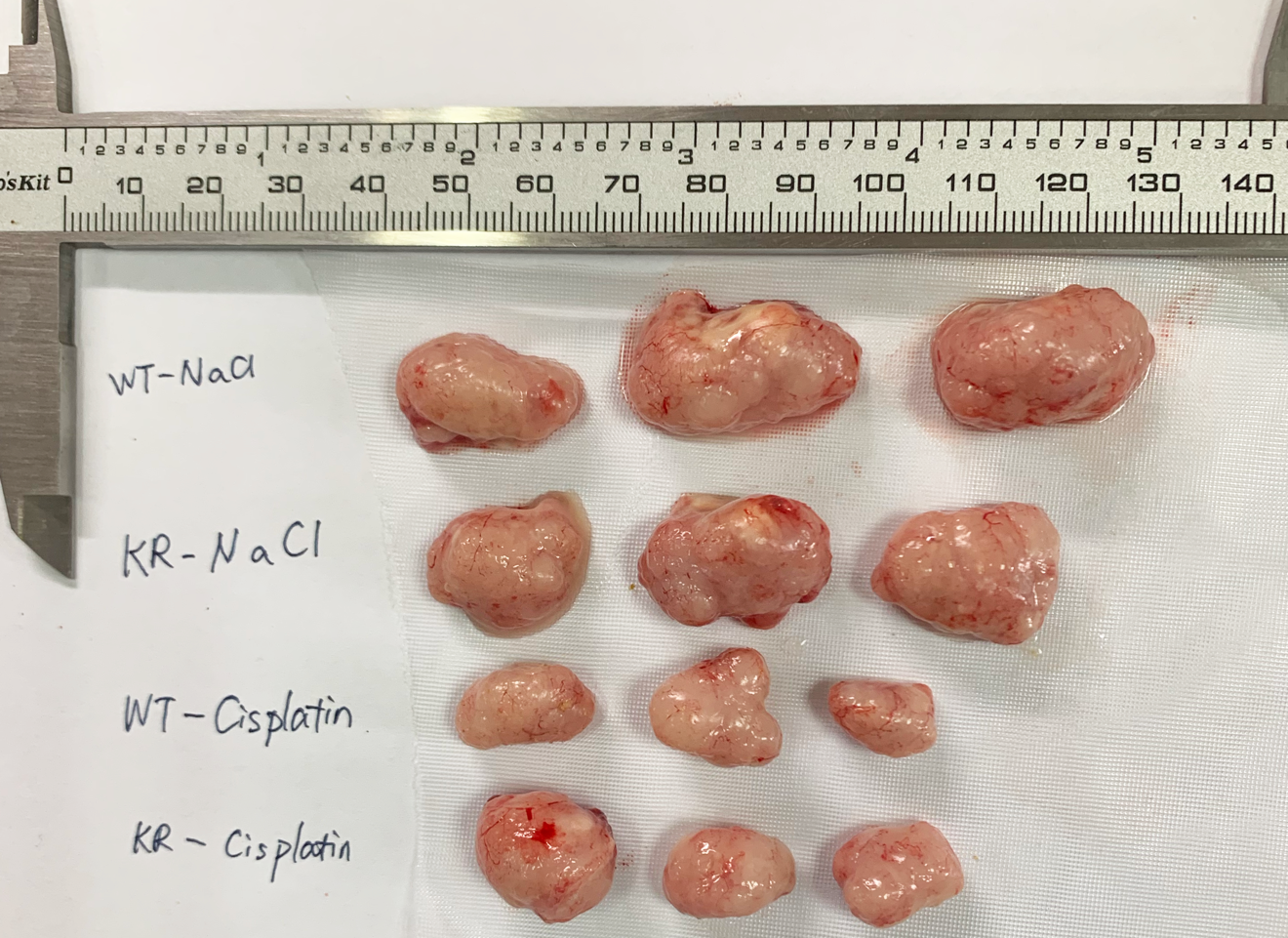


**Supplementary Fig. S4. Tumor presentations in killed mice.**

Tumors were harvested from xenografts of groups of HGC27 CCNC^–/–^+CCNC^3KtoR^ and CCNC^–/–^+CCNC^WT^ with or without cisplatin administration.


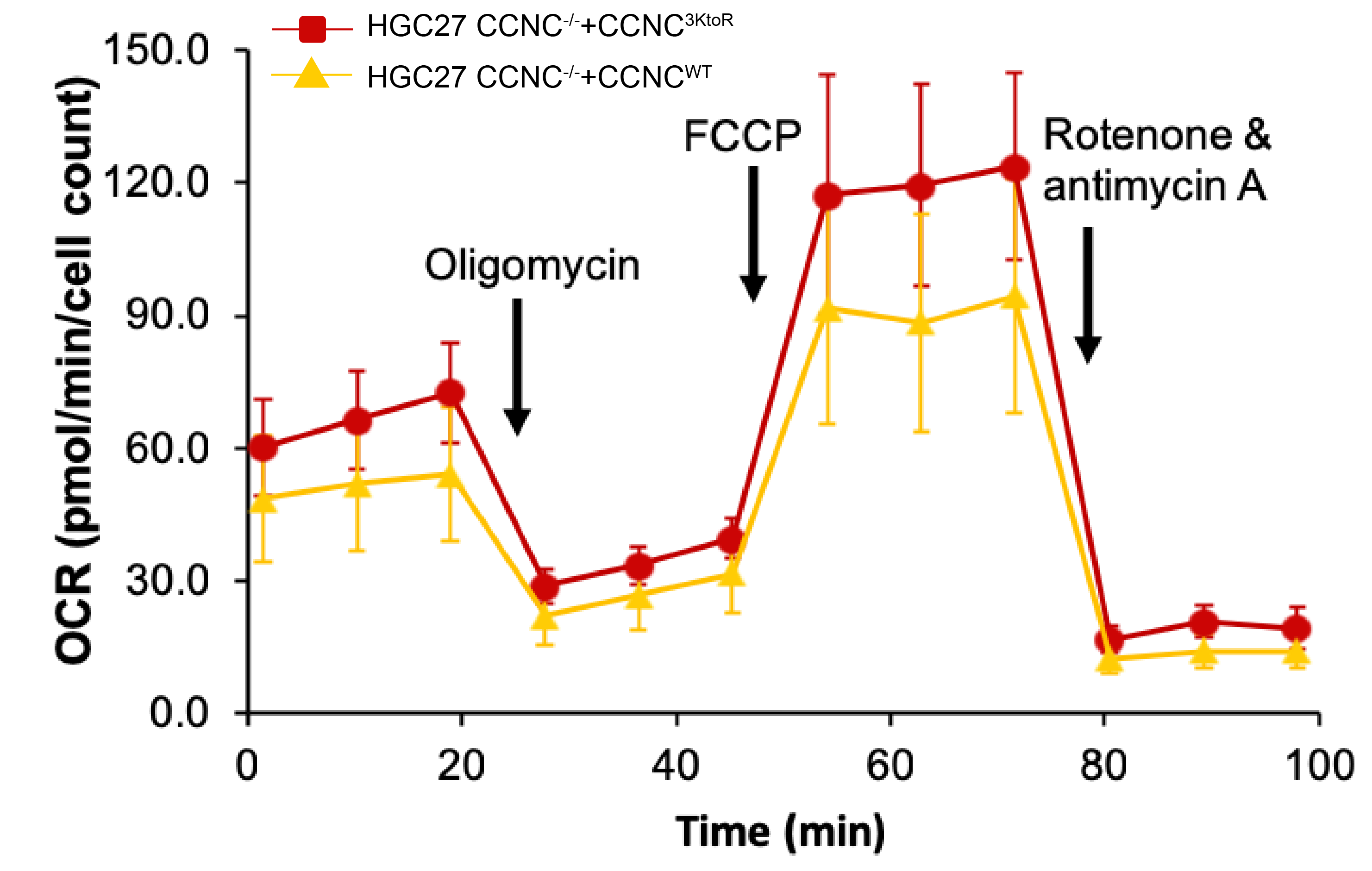


**Supplementary Fig. S5. Oxygen consuption rate in HGC27 CCNC^–/–^+CCNC^3KtoR^ and CCNC^–/–^+CCNC^WT^**

HGC27 CCNC^–/–^+CCNC^3KtoR^ and CCNC^–/–^+CCNC^WT^ were treated with cisplatin for 24h and then detected for oxygen consumption rate

**Table S1. Plasmids used in this study.** (docx)

| Figure1 | Figure2 | Figure 3 |
| --- | --- | --- |
| pCDNA3.1-CCNC-HA | pET22b-UBA1 | pLenti-Crispr-HACE1 |
| pCDNA3.1-HACE1-Flag | pET22b-UBCH7 | pLenti-Crispr-CCNC |
| pET22b-GST-HACE1 | pCDNA3.1-CCNC-Flag | pCDH-CCNC-Flag |
| pGEX4t-1-HACE1(1-257) | pACYC-HA-Ub-UBCH7-UBA1-HACE1 | pCDH-CCNC-Flag-3KtoR |
| pGEX4t-1-HACE1(258-909) | pACYC-HA-Ub-UBCH7-UBA1 |  |
| pGEX4t-1-HACE1(1-546) | pET22b-CCNC-His6 |  |
| pGEX4t-1-HACE1(547-909) | pcDNA3.1-HACE1-Myc |  |
| pDEST32-CCNC | pRK5-HA-Ub(K6) |  |
| pDEST32-HACE1 | pRK5-HA-Ub(K11) |  |
| pDEST22-CCNC | pRK5-HA-Ub(K27) |  |
| pDEST22-HACE1 | pRK5-HA-Ub(K29) |  |
|  | pRK5-HA-Ub(K33) |  |
|  | pRK5-HA-Ub(K48) |  |
|  | pRK5-HA-Ub(K63) |  |
|  | pCDNA3.1-CCNC-K126R |  |
|  | pCDNA3.1-CCNC-K226R |  |
|  | pCDNA3.1-CCNC-K236R |  |
|  | pCDNA3.1-CCNC-K126, K226R |  |
|  | pCDNA3.1-CCNC-K126R, K236R |  |
|  | pCDNA3.1-CCNC-K226R, K236R |  |
|  | pCDNA3.1-CCNC-K126R, K226R,K236R |  |
|  | pRK5-HA-Ub |  |
|  | pCDNA3.1-HACE1-Flag |  |

1. Liu, Z. *et al.* Ubiquitylation of autophagy receptor Optineurin by HACE1 activates selective autophagy for tumor suppression. *Cancer Cell* **26**, 106-120 (2014).

2. Xu, X. *et al.* Excessive UBE3A dosage impairs retinoic acid signaling and synaptic plasticity in autism spectrum disorders. *Cell Res* **28**, 48-68 (2018).

3. Yang, G. *et al.* Proteomic, functional and motif-based analysis of C-terminal Src kinase-interacting proteins. *Proteomics* **9**, 4944-4961 (2009).

4. Rappsilber, J., Ishihama, Y. & Mann, M. Stop and go extraction tips for matrix-assisted laser desorption/ionization, nanoelectrospray, and LC/MS sample pretreatment in proteomics. *Anal Chem* **75**, 663-670 (2003).

5. Liu, H. & Naismith, J.H. An efficient one-step site-directed deletion, insertion, single and multiple-site plasmid mutagenesis protocol. *BMC Biotechnol* **8**, 91 (2008).
